# Effect of Sharp Jumps at the Edges of Phase Response Curves on Synchronization of Electrically Coupled Neuronal Oscillators

**DOI:** 10.1371/journal.pone.0058922

**Published:** 2013-03-29

**Authors:** Ramana Dodla, Charles J. Wilson

**Affiliations:** Department of Biology, University of Texas at San Antonio, San Antonio, Texas, United States of America; University of Pittsburgh, United States of America

## Abstract

We study synchronization phenomenon of coupled neuronal oscillators using the theory of weakly coupled oscillators. The role of sudden jumps in the phase response curve profiles found in some experimental recordings and models on the ability of coupled neurons to exhibit synchronous and antisynchronous behavior is investigated, when the coupling between the neurons is electrical. The level of jumps in the phase response curve at either end, spike width and frequency of voltage time course of the coupled neurons are parameterized using piecewise linear functional forms, and the conditions for stable synchrony and stable antisynchrony in terms of those parameters are computed analytically. The role of the peak position of the phase response curve on phase-locking is also investigated.

## Introduction

A phase response curve (PRC) quantifies temporal deviations of an oscillator in response to an oncoming stimulus [1]–[3]. Methods based on PRCs have been extensively used to predict when synchronization could occur between biological oscillators [4]–[7]. Studies based on weakly coupled oscillator theory applied to coupled neurons have often sought to relate the shape of the PRC to the emergence and the stability of the phase-locked states [8]–[14]. However, some PRCs measured experimentally exhibit significant departures from being smooth and continuous [15]–[18]. In particular these PRCs may show sudden increments in their level at the beginning and/or end of the oscillation cycle. There are also some neuronal models that display such behavior. Such sudden increments in the PRC level are often due to fast gating dynamics of the neurons, but could also be due to the effect of higher order PRCs that may become significant in the collective dynamics of coupled neurons. If the sudden rise in the PRC level is indeed due to fast gating dynamics, then, for simplicity, it could rather be approximated by a discontinuous jump in the PRC. When higher order PRCs become significant, then their shapes may also have to be incorporated in the relevant analysis.

Sharp jumps in the PRCs were earlier shown to be caused by fast potassium gating dynamics [19], presence of adaptation currents such as calcium-dependent afterhyperpolarization (AHP) current or muscarinic voltage-dependent potassium (M) current [20], [21], or abrupt dynamical changes in the modeling equations. AHP current, for example, can cause the neuron become less sensitive at the early phases and thus impart skewness to the PRC. In some models it can also impart sudden jumps at early phases [21].

The PRCs of a leaky integrate-and-fire model [22] and quadratic integrate-and-fire model [23], [24] display discontinuities at the beginning and the end of the oscillation cycle. Adapted exponential integrate-and-fire neuron model [21], [25] is another example that displays sharp PRC jumps. In all these cases weakly coupled oscillator theory has been used to predict the stability of synchrony and antisynchrony. As we will also illustrate, adaptation is not necessary to realize PRC with sharp jumps. But even when adaptation was present, we expect that the theory would still be applicable [20] because the effect of adaptation on synchrony is via a modification of the shape of the PRC, and/or the voltage time course; In the synchronized state the change of frequency caused by adaptation can be assumed to be negligible.

Here we address comprehensively the role of the discontinuous jumps at the ends of the PRCs in the synchronizability of coupled oscillatory neurons when the coupling between them is weak and electrical. Only the first order PRC is used in the analysis. To model the phase response curve, we employ a piecewise linear approach that allows a detailed study of the dependence of the PRC shape on synchrony, while at the same time being applicable to experimentally determined PRCs. The PRC profile is constructed with only two piecewise linear segments, and the voltage profile with three piecewise linear segments. We predict when synchrony and antisynchrony become stable as the level of the discontinuous PRC jumps, and the spike width and frequency are varied. Our study complements other similar studies on electrically coupled neuronal networks that used leaky integrate-and-fire models [12], [26], and generalizes the results of those that used quadratic integrate-and-fire models [23], [24] by considering a range of PRC shapes and voltage time courses. We also study how the location of the PRC maximum (the skewness) affects synchrony and antisynchrony. A network simulation of Wang-Buzsáki model neurons when each neuron displays a PRC with sudden rise in its level near zero-phase is also presented.

## Model

**Figure 1:**
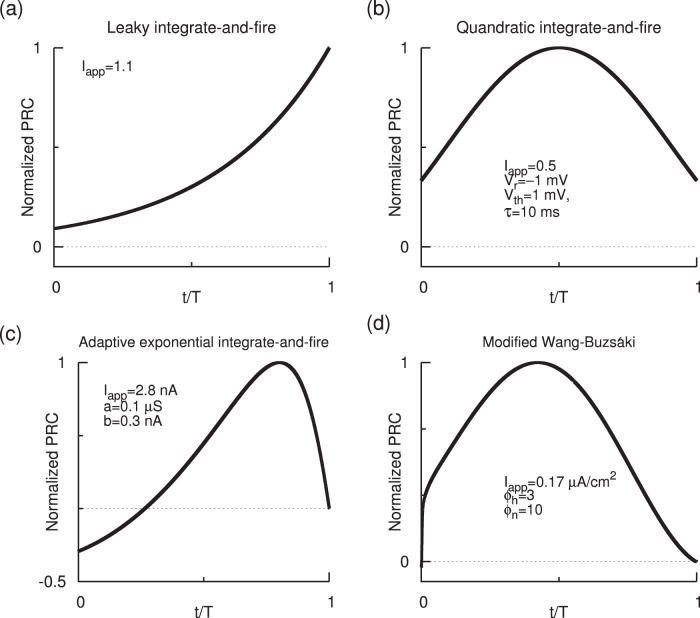
Prevalence of sharp jumps in the PRCs in some neuronal models. (a) PRC of leaky integrate-and-fire neuron model [22], [35]: 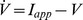 where 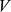 is reset to 0 when it crosses a threshold level of 1 (in normalized units). (b) PRC of quadratic integrate-and-fire neuron model [23]: 
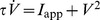
 where *V* is reset to 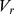 when it crosses a threshold level 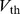. (c) PRC of adaptive exponential integrate-and-fire model [21], [25]: 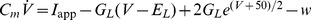, 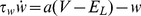 such that *V* and 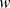 are reset, respectively, to 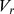 and 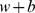 whenever 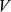 reaches a peak level 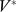. The parameters are 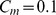 nF, 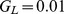 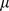
S, 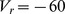 mV, 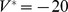 mV, 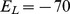 mV, and 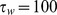 ms. The level of adaptation is controlled by 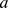. (d) PRC of Wang-Buzsáki model that is described in the Model section. Models in (a, b, d) have no adaptation and their second and higher order PRCs are identical to that of the first order.

The leaky integrate-and-fire, quadratic integrate-and-fire, and adaptive exponential integrate-and-fire models whose PRCs are depicted in Fig. 1(a–c) are described in the figure caption. The modified Wang-Buzsáki model [19] whose PRC is depicted in Fig. 1(d) is given by the following evolution equations:
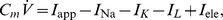


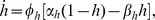


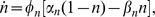
where 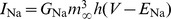, 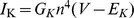, 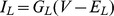, 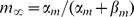, 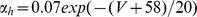, 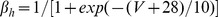, 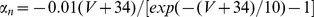, and 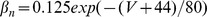. The parameters are 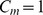 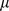
F/cm^2^, 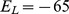 mV, 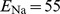 mV, 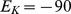 mV, 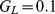 mS/cm^2^, 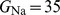 mS/cm^2^, and 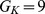 mS/cm^2^. The applied current 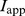 when set above 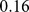 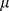
A/cm^2^ triggers spontaneous oscillations. Ignoring a very brief downward and negative swing near zero phase, the PRC of the model may be considered a type-1 and may be treated like a PRC with sharp jump at the left edge [Fig. 1(d)]. The only difference between our formulation and the original formulation of this model is in making the time-scale factor for sodium inactivation and potassium activation independent: 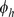 and 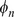 that are now used to control the PRC shape. This model is also used later in network simulations where the current due to electrical coupling 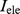 is proportional to the difference of the voltage of the coupled neurons.

**Figure 2:**
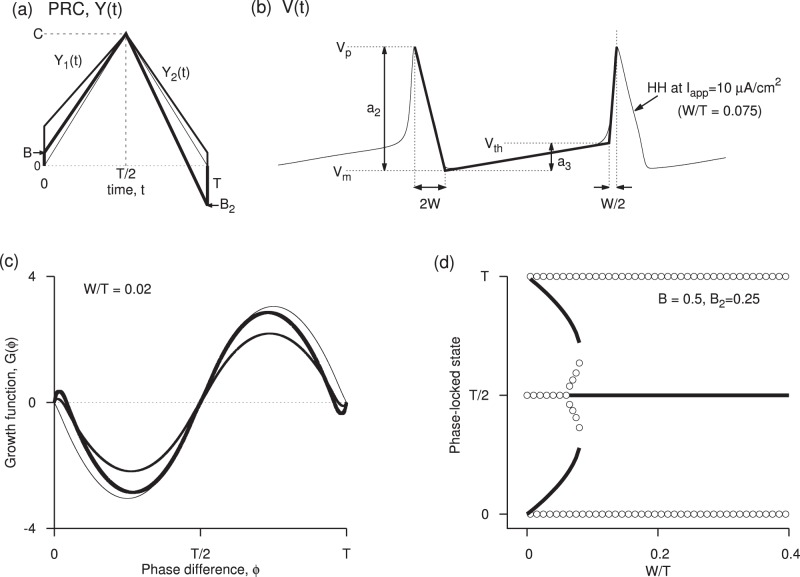
Piecewise linear models of PRC and voltage, and illustration of spike width effect. (a) Sample piecewise linear PRC profiles studied. (b) Voltage time course that consists of three piecewise linear profiles, modeled after the classic Hodgkin-Huxley model. (c) Growth function 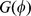 computed for the three PRCs displayed in (a) and the voltage time course in (b). The sharp drops of the PRC at the edges altered the stability of synchrony. (d) Bifurcation diagram as a function of the normalized spike width at 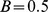 and 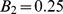. In this and later figures, solid lines indicate stability and open circles instability of the phase-locked solutions. Synchrony is unstable at all frequencies, whereas antisynchrony is stable at high frequencies.

The main results of the paper use the formulation laid out below. Extension of this model that incorporates PRC skewness is presented in the later part of the Results section. We formulate the PRC and the voltage time course using piecewise linear (PWL) functions, and then present a method to find the stability of synchrony and antisynchrony. The choice of PWL functions facilitates analytical determination of stability boundaries. The phase response curve is formulated as a function with two piecewise linear profiles [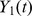 and 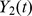] that exhibit finite jumps or discontinuities at the edges (i.e. at 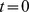 and at 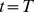, the period of oscillation of the neuron). The assumption of finite jumps at the edges is not necessarily due to a discontinuity in the PRC, but sharp rise or fall at those phases. But for computation of stability of phase-locked solutions, the assumption of a discontinuous jump at the edges does not lead to any artifacts unless another parameter such as spike width also simultaneously becomes zero; In such a case the effect of discontinuity must be explicitly incorporated into the analysis, or the analysis must be carried out in the limit of those parameters going to zero. The PRC is formulated as below:
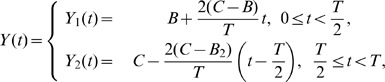
(1)where 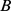 is the amount of jump on the left edge (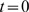), 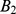 is the amount of jump on the right edge (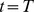) of the PRC, and 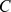 (>0) is the maximum advancement of the PRC. When 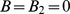, the PRC becomes symmetric. The PRC profile is depicted in Fig. 2(a) for a few parameters of 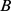 and 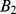. A monotonically increasing PRC as in a leaky integrate-and-fire model is obtained by setting 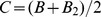. Such PRCs are also treated in the Results section as a special case. No assumption is made on the sign of 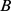 and 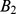 in deriving the stability regions.

The voltage profile is formulated by the following three piecewise linear curves [Fig. 2(b)] that are modeled after an empirical observation of the voltage profile of the Hodgkin-Huxley (HH) model equations; Spike width parameter 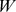 is the only time varying parameter that appears in the model, and the spike amplitude is controlled by spike peak 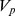, spike threshold 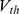, and spike minimum, 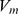:
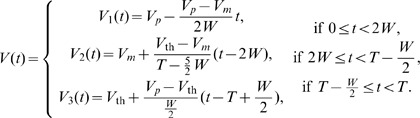
(2)


At an external applied current of 10 *µ*A/cm^2^, the HH voltage time course can be approximated with 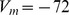 mV, 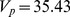 mV, 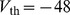 mV, and 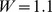 ms, 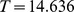 ms (i.e. an oscillation frequency of 68.3 Hz). Thus 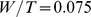 for this model. We define 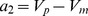, and 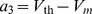. We term 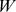 the spike width for simplicity although the actual spike width could be up to 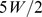. The spike width and threshold to spike height ratio (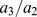) are freely altered to explore the stability boundaries in these parameter spaces. But we assume that
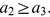
(3)


Because the spike downstroke and spike upstroke together add up to a width of 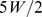, we insist that *T* is bigger than that. That is,
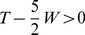
(4)


Pairs of identical nonlinear oscillatory neurons that may be originally described by several state variables, but oscillate with a constant period *T* and possess a voltage profile 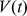, and a PRC, 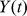, when coupled electrically by a coupling function proportional to the difference of their individual voltages can be reduced to pairs of phase evolution equations under the assumption of weak coupling [1]–[3], [27]–[31]. Two such identical neurons can be reduced to two phase evolution equations with phases 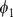 and 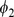 (that range from 0 and *T*) described by the following two equations
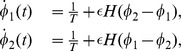
where 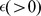 is the strength of coupling. 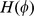 is the interaction function and quantifies the instantaneous increase of an oscillator frequency due to coupling to the other oscillator, and is expressed as follows:



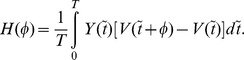



Phase-locking occurs when the phase difference 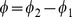 remains constant in time, and hence it is convenient to study the equations in terms of the phase difference that evolves according to
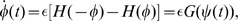
(5)where



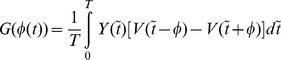
represents the growth function that quantifies the instantaneous growth of the phase difference. The change of the growth function as a function of the phase difference [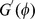] at a stable phase-locked state (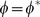) should become negative so that any small perturbation to that steady state subsides. Thus the stability of the synchronous state (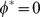) of the Eq. 5 is determined by the eigenvalue:
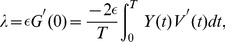
(6)and the stability of the antisynchronous state (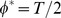) of the Eq. 5 is determined by the eigenvalue:



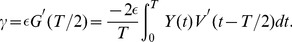
(7)These eigenvalues are computed for the 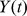 and 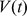 profiles formulated in Eqs. 1 and 2, and the stability of synchrony and antisynchrony is determined in the following sections. We will see that the PRC jumps (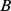 and 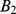) can alter the stability of both synchrony and antisynchrony. A symmetric PRC [Fig. 2(a), thin curve] leads to stable synchrony and unstable antisynchrony [Fig. 2(c), thin curve]. An example of the synchronous state becoming unstable and the emergence of a non-zero phase-locked state that is very close to the synchronous state due to the finite jumps at the PRC ends is shown in Fig. 2(a,c). Antisynchrony and other non-zero phase-locked states can also undergo stability changes. The case of zero spike width (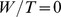) presents an enigmatic situation when the unstable synchronous branch and a stable non-synchronous but phase-locked branch converge at the same point [Fig. 2(d)]. This situation arises because of the fact that the discontinuity in the voltage due to 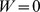 and the discontinuous jump in the PRC occur at the same temporal location. The stable phase-locked branch is very close to the unstable synchrony branch, and they quickly get separated as 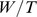 is increased.

Hence strictly at 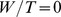, the stability criterion for phase-locked states depends on whether it is computed in the limit of 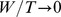 or otherwise. We will compute the stability of synchrony and anti-synchrony in the limit of 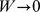 that inherently uses the spike effect, and will see a transition from stable synchrony to unstable synchrony above a critical 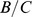. For non-zero 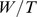, this situation does not arise because the edge effects get factored into the eigenvalue components computed due to the up and downstrokes that span finite time widths. The antisynchronous state is unstable for 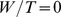 unless the sum of the PRC jumps is bigger than twice the PRC maximum (i.e. 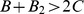). But as 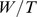 increases, more parameter region is filled with antisynchrony, some with synchrony, and some with bistability. The bistability could occur between phase-locked states, synchronous, and antisynchronous states.

The stability boundaries do not depend on the time period 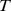, but in numerical simulations we used the period corresponding to the HH model mentioned earlier. The other spike parameters are also derived from the same model.

## Results

**Figure 3:**
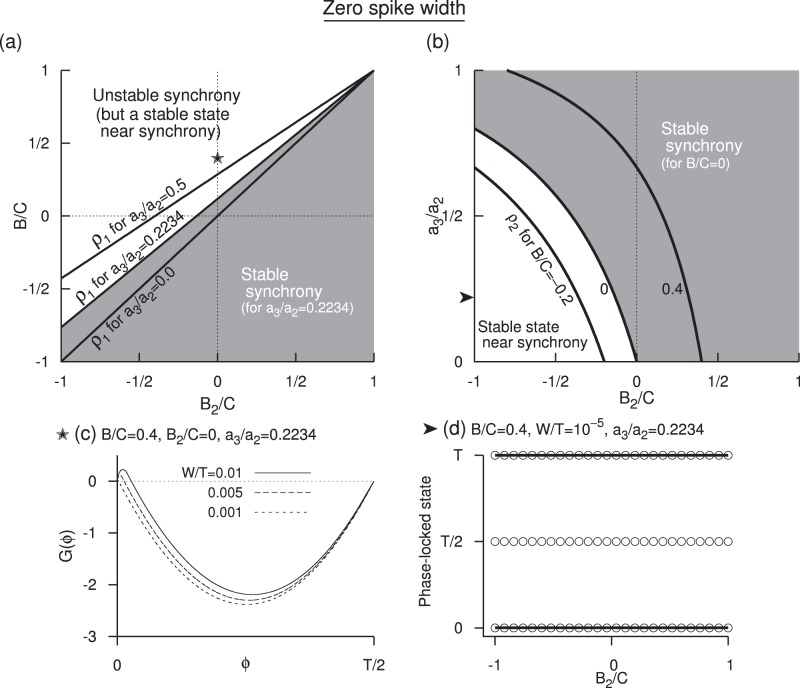
Edge effects when spike width is zero. (a) Stable synchrony (shaded region) and the unstable synchrony (white region) for 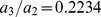 in the plane of 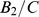 and 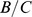. Boundary curves for two other levels of 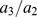 are also shown. (b) Same as in (a) but in the plane of 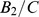 and 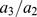 for 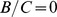. Boundary curves for two other level of 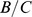 are also displayed. These curves are obtained by inverting equation 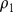 for 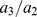. Antisynchrony is unstable in the displayed parameter ranges in (a) and (b). (c) Growth function 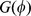, in the limit of zero spike width, displaying unstable synchrony but a stable phase-locked state that is very close to the synchronous state for a parameter value that is in the unstable synchrony region. (d) One-parameter bifurcation diagram as a function of 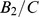.

**Figure 4:**
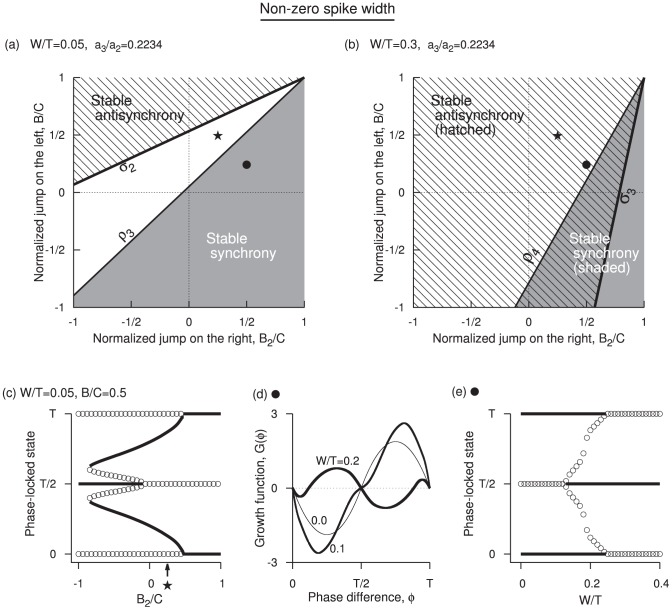
Edge effects when spike width is non-zero. (a) Stable synchrony (shaded) and stable antisynchrony (hatched) regions in 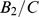 and 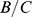 space for small spike width 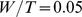 at 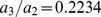. The white region holds other non-zero stable phase-locked solutions. (b) Same as in (a) but for large spike width 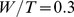. (c) One-parameter bifurcation diagram as a function of 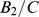 at small spike width and 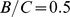, and 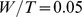. The non-zero phase-locked states are found to be bistable with anti-synchronous state, and are not very close to the synchronous state as was the case for zero spike width. (d) Growth function at three levels of 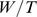 when 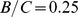 and 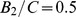. When 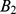 and 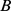 are chosen such that the system is in a synchronous state, increasing spike width eventually makes it unstable, but the stability is maintained until the spike width is large. (e) One-parameter bifurcation diagram as a function of spike width corresponding to the parameters in (d). The antisynchrony becomes stable here at 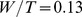. Note that the stability boundaries and transitions are functions of the ratios 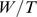, 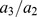, and 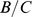. Thus, for example, the diagram in (a) is valid for any period and spike width as along as 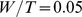.

**Figure 5:**
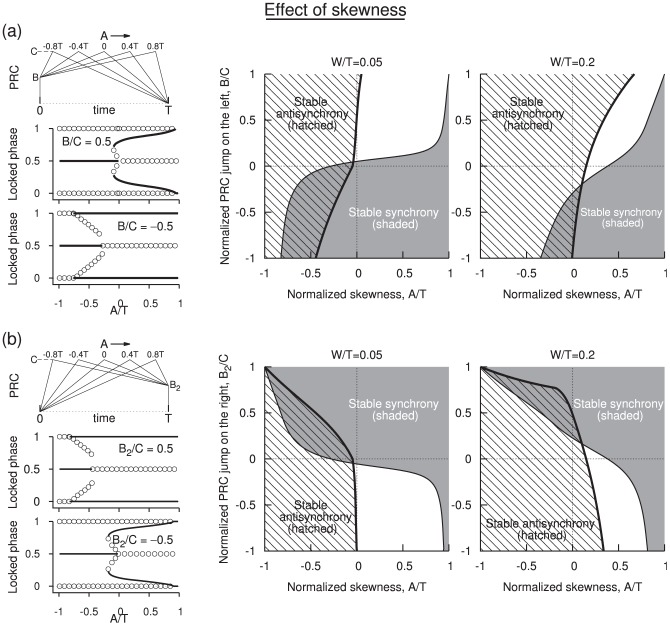
Effect of skewness on the PRCs that have left side jump (a), and those that have right side jump (b). The left jump PRCs are parametrized by 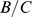 and the right jump PRCs are parameterized by 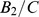. (a) A sample set of PRCs as the skewness 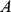 is moved from negative to positive levels is illustrated in the left column along with one-parameter bifurcation diagrams at two different levels of 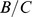 as the skewness is increased (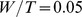). For positive jump synchrony is mostly unstable, and at large skewness even the antisynchrony is destabilized. But for negative jump skewness helped stabilize synchrony. On the right parameter planes depicting stability regions in the plane of skewness and jump are illustrated at different levels of 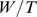. (b) Same as in (a) but for right side jump PRCs (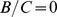). 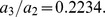

Except the leaky integrate-and-fire (LIF) model which has an exponential form of PRC, the other three PRCs [quadratic integrate-and-fire (QIF), adaptive exponential integrate-and-fire (aEIF), and modified Wang-Buzsáki model] presented in Fig. 1 can be approximated by PWL formulations in our models. The results will also be applicable to the LIF model in a qualitative manner. The LIF and QIF models both have sharp jumps in the PRC at both early and late phases, and no adaptation currents are present in either model. The aEIF model has an adaptation current variable and shows a left PRC jump. The modified Wang-Buzsáki model that has only sodium and delayed rectifier currents, and no adaptation currents, displays a sudden downward swing followed by a sudden rise in the PRC level. We first study symmetric PRCs depicted in Fig. 2(a). Rigorous analytical arguments are presented for the boundaries of both stable synchrony and stable antisynchrony (Figs. 3 and 4) when the PRC is symmetric (Eq. 1, Fig. 2). Later we introduce PRC skewness that parametrizes the position of the peak PRC level, and analytical results are discussed (Fig. 5) when the PRC in addition acquires a skewness (Eq. 16) such that the peak of the PRC is either moved to the left or right. Finally we present simulation results using networks of Wang-Buzsáki model.

### 1. Synchrony and Antisynchrony when Spike Width is Zero

The boundaries of synchrony (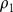 and 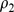) and antisynchrony (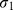) under the assumption of zero spike width are derived in this section, and are illustrated in Fig. 3(a,b) in the parameter spaces of 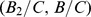 and 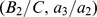. In the absence of any jumps in the PRC, the coupled system synchronizes because the entirely positive PRC encounters a positively sloped voltage segment and thus the eigenvalue [Eq. 6] becomes negative. In other words, if the second neuron leads the first by a small phase, the first neuron speeds up in response because the voltage time course of the second neuron (say, 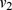) is higher than the first (say, 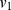) for most of the spike interval (coupling to the first neuron is proportional to 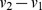). And if the second neuron lags the first, the converse effect occurs.

A positive jump in the PRC at zero phase (left side edge) helps destabilize synchrony, and a positive jump at 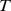 (right side edge) helps stabilize synchrony more. We can visualize these effects by imagining the case of non-zero spike width. The spike downstroke has large negative slope, and the spike upstroke has large positive slope. Since the PRC is non-zero, the convolution (Eq. 6) with the downstroke results in a positive eigenvalue that helps destabilize synchrony, and that with the upstroke results in a negative eigenvalue that helps stabilize synchrony. In other words, if 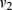 leads 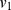, the first neuron slows down in the region from spike peak to (nearly) the spike downstroke because the spike downstroke of 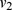 falls below that of 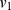, whereas it speeds up during the spike upstroke region because there 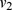 is bigger than 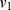. In the limit of zero spike width, these effects remain because the PRC is non-zero due to finite jumps. Thus the destabilizing effect of the left edge (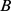) can be countered by appropriately increasing the right side jump (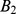) leading to a diagonal line of criticality in the parameter space of 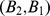
[Fig. 3(a)]. Increasing the spike threshold such that 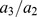 is also increased will cause the voltage time course acquire more positive slope that enhances the negative eigenvalue component, and consequently we see the boundary of synchronous region being pushed into the earlier unstable region [Fig. 3(a)] or making a transition to synchrony [Fig. 3(b)] with increasing 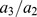.

The instability of synchrony occurring for 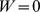 is more subtle than that occurring for non-zero spike width. Imagine again the case of non-zero spike width. When 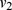 leads 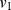, the slow down of the first neuron occurs within the duration of spike downstroke, and then it speeds up for the rest of its cycle. When the spike width is zero, the slow down regime is really confined to zero width, but it still exists because of the non-zero value of the PRC at the edge. Thus the turn around from speed up to slow down occurs right at the zero phase, thus creating an equilibrium point (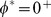) that becomes stable when the synchrony becomes unstable. The growth function displaying a stable non-zero equilibrium (due to the negative slope) merging with unstable synchronous state (due to positive slope) as the spike width becomes zero is illustrated in Fig. 3(c). Similar phenomenon occurs even in the stable synchrony regime, leading to a stable synchrony coexisting with unstable equilibrium point (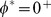) [Fig. 3(d)]. Thus the boundaries of synchrony derived below are in the limit of 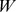 going to zero. And for very small non-zero 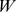 these boundaries begin to change slightly in the parameter spaces, and the non-zero equilibrium and the synchronous states become more separated.

If the PRC is maximum at half period, the time-shifted (by half period) voltage not only has a large negative discontinuity, but also encounters a large PRC level at the discontinuity. Together this contributes to large positive integral in the eigenvalue of the antisynchrony (Eq. 7). In other words, if 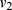 leads 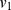, 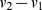 that drives the first neuron becomes large negative at half period, and thus the first neuron slows further. If this effect is not countered by the other segments of the voltage (which occurs when condition in Eq. 10 is satisfied), the antisynchrony remains unstable.

The eigenvalue for the synchronous state (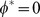) is obtained by using the formula in Eq. 6, and then adding the edge contributions from both the jumps at the end. Using the formula in Eq. 6 we get two eigenvalue components, 
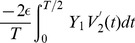
 and 
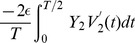
 which when evaluated and added yield 
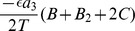
. The edge contribution from the left side jump of the PRC is 
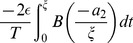
 which when evaluated in the limit of 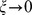 yields 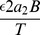. Similarly carrying out such an integral at the right side jump of the PRC yields 
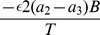
. Adding all these four eigenvalue components results in the total eigenvalue that determines the stability of the synchronous state: 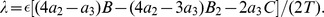 We directly see that a positive left side jump makes the eigenvalue more positive and hence making it less likely to synchronize, and a positive right side jump makes the eigenvalue more negative and hence making it more likely to synchronize. The maximum PRC advancement works in favor of synchrony. The synchronous state is stable when the eigenvalue is negative, i.e. when
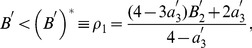
(8)where 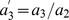, 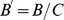, and 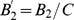. The region of synchrony bounded by the curve 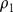 is illustrated in Fig. 3(a) for three levels of 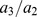. The above critical condition can also be written in terms of the ratio 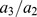 that gives more convenient way to visualize the stability region as a function of the level of spike threshold:



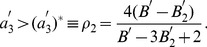
(9)The stable synchrony region bounded by 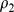 is illustrated in Fig. 3(b) as a function of the jump at the right edge at three levels of the left edge jumps.

Next we find the stability conditions for antisynchrony. Since 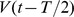 is discontinuous at 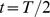, the discontinuity computed as before results in an eigenvalue component that is equal to 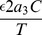. The other two eigenvalue components are identical to those derived for the case of synchrony. Combining all the three components, the total eigenvalue is obtained as 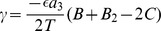. The antisynchronous state becomes stable when this eigenvalue becomes negative, i.e. when 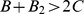, or when
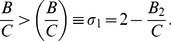
(10)


For most PRCs that may have jumps, this condition may be difficult to satisfy because it requires large drops at the edges that cumulatively exceed the maximum PRC advancement found at 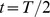. The region falls outside the depicted range of 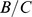 in Fig. 3(a), and thus the depicted range holds an unstable antisynchronous state. The stability is independent of 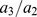.

### 2. Non-zero Spike Width and Effect of Frequency

Boundaries of synchrony (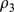 and 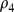) and antisynchrony (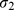 and 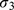) in the presence of non-zero spike width are derived in this section. For non-zero 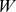, the non-zero equilibrium and the synchronous state that were found merging get separated as seen in Fig. 3(c). The slopes of the spike downstroke and upstroke are proportional to the spike width, and hence their contributions to the growth of the phase difference also is proportional to 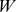 for small 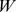. Consequently, the resultant boundaries for small non-zero 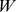 are near those obtained for zero spike width. As in the case of zero spike width, the PRC jump on the left diminishes the chances of synchrony, and that on the right promotes the chances of synchrony. Correspondingly, large PRC jump on the right and small jump on the left signifies stable synchronous region, and large jump on the left side and small jump on the right destabilizes the synchronous state [Fig. 4(a)].

The antisynchronous state was unstable in most of the parameter space at 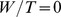 due to the destabilizing effect of the voltage discontinuity of 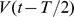 at 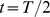. With increasing 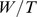, the upstroke broadens at a lower rate than the downstroke, and consequently the stabilizing effect of the upstroke dominates resulting in boundary of the stable antisynchrony becoming sensitive for 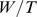. At large 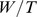, most of the parameter space is filled with antisynchronous state [Fig. 4(b)]. Unlike the case of zero spike width, the stable near-zero phase-locked state and the synchronous state are well separated [Fig. 4(c)], and a bistability between a non-zero phase-locked state and the antisynchronous state is found for large left PRC jumps. Increasing the frequency causes bistability between synchronous and antisynchronous states [Fig. 4(d)], before the synchrony loses stability [Fig. 4(e)].

#### Synchrony

As the spike width becomes bigger than zero, the edge effects that we had to include earlier (when 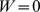) are naturally contained in the contributions of the up and downstrokes of the spike. Apart from these two components, the regime from the spike minimum to the PRC maximum, and PRC maximum to the spike threshold provide the other two components to the integral in Eq. 6. But if the spike minimum occurs after 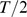, then the downstroke contribution extends all the way up to 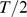, and the other three components are contained in the regime 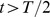. When the spike width is small such that 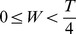, we obtain the region of stable synchrony as (see Methods)
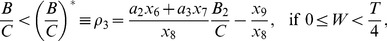
(11)where 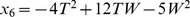, and 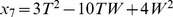, 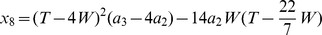, and 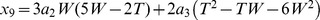. The parameters 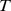 and 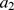 can be used to further normalize 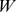 and 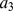 respectively. The region of stable synchrony bounded by 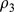 is illustrated in Fig. 4(a) for 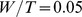 and 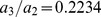.

And when the spike width is large (or the frequency is high) such that the spike minimum occurs after the PRC peak (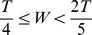), the region of stability of synchrony is given by (see Methods).
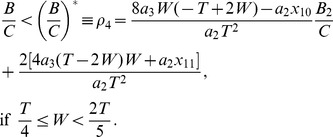
(12)where 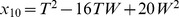, and 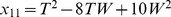. The parameters 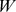 and 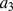 can be normalized, respectively with 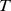 and 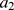. The stable synchrony region bounded by 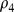 is illustrated in Fig. 4(b) for 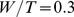 and 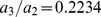.

#### Antisynchrony

The effect due to discontinuity of 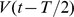 at 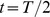 that existed for zero spike width is now contained in the 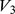 and 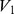 segments that fall on either side of 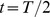. Along with these two segments, the segment preceding the spike peak and the segment that follows the spike threshold contribute to the integral in Eq. 7. When the spike width is small such that 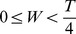, we obtain the region of stability for antisynchrony as (see Methods)
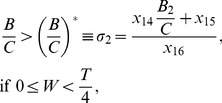
(13)where 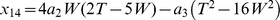, 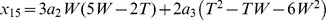, and 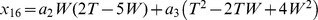. The stable antisynchronous region bounded by 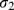 is illustrated in Fig. 4(a) at 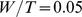 and 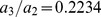 (that corresponds to the HH model).

When the spike width is large such that 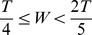, the spike downstroke effect lasts from 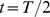 to 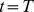, and the other three segments fall in the first half of the period. The corresponding boundary for stable antisynchrony is more complex than before, and is given in two regimes of spike width (see Methods). The stable antisynchrony exists when
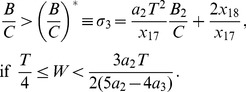
(14)When 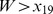, we arrive at the region of stable antisynchrony as



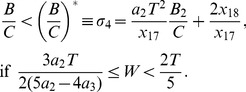
(15)At 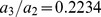 the limits for the curves 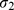 and 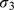 are given respectively by 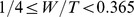 and 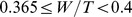. At 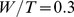 only 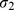 exists, and the region bounded by it is illustrated in Fig. 4(b).

### 3. Effect of PRC Skewness

The location of the PRC peak could indeed affect the onset of synchrony and antisynchrony. We introduce the skewness by redefining the two PRC segments of Eq. 1 as below.
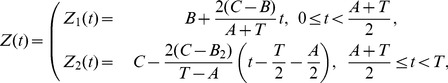
(16)where the parameter 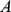 is the skewness parameter and could range from 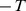 when the maximum PRC advancement occurs at zero-phase to 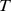 when the same occurs at phase 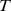. The shapes of the PRCs are illustrated in two special cases when only the left side jump is present [Fig. 5(a) left top] or when only the right side jump is present [Fig. 5(b) left top]. The other panels in the figure present corresponding transitions of stability as the level of these jumps and the skewness are altered. The one-parameter bifurcation diagrams are computed numerically, and the stability boundaries presented are derived from the expressions obtained by solving the eigenvalue equation as was done in the previous sections.

For PRCs that have jumps only on the left side an example of transition of stability between phase-locked states is shown in Fig. 5(a, left middle) for small spike width. In this illustration the level of the jump is set at half of the peak PRC level. Synchrony is unstable for most of the skewness, except in a small window at very large skewness. For negative skewness the level of 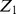 during the spike downstroke is bigger than that at zero skewness, and thus the positive eigenvalue component contributed by the downstroke increases resulting in a loss of stable synchrony. However, at very large skewness the destabilizing effect of the downstroke can be countered by the enormously negative eigenvalue contributed by the spike upstroke in association with the increasing level of 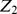 segment, thus giving a thin regime of stable synchrony [Fig. 5(b, right panel 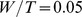)]. Increasing 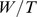 increases this regime because now more length of the PRC segments fall inside the up and downstroke regimes; the upstroke having a bigger slope contributes a dominating eigenvalue [Fig. 5(a, right panel 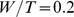)]. An example of transition of stability is shown for negative 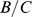 in Fig. 5(a, left bottom). There is no sudden transition as 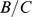 goes from positive to negative regime. The stability curves are continuous. For negative 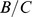 a portion of the spike downstroke contributes to stable synchrony due to negative 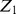 segment, and thus stable synchrony can be sustained even for large negative skewness. In the previous section we saw that it is possible to make the antisynchronous state stable by increasing the spike width or frequency. This advantage is now countered by a positive skewness; Now the downstroke that is responsible for instability dominates because the entire (half-period shifted) spike profile encounters an increasing segment 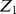. Consequently the PRC level during the downstroke is higher than that during upstroke. Thus it is difficult to attain stable antisynchrony for large skewness [Figs. 5(a, right panels)].

The corresponding results for a right side jump of the PRC are illustrated in Fig. 5(b). For synchronous and antisynchronous states, the parameter 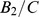 behaves qualitatively like 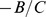, i.e. the right side jump is qualitatively equivalent to a left side jump with the opposite sign, and vice versa [panels in Fig. 5(b)]. This equivalency seems better at low frequencies or spike widths, i.e. when 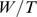 is very small. We can indeed determine when this equivalency is a valid approximation by examining the eigenvalues that define the stability of synchrony and antisynchrony. For ease of analysis, we examine the case when the skewness is absent (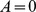). The eigenvalue under study is 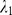 (see Methods), and for showing the exact equivalency we must show that the dependence on 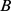 (i.e. the coefficient of 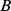) is the same as the dependence on 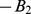 (i.e. the negative of the coefficient of 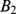). In other words, the sum of the coefficients of these terms must be zero. The sum of the numerators of these two coefficients is proportional to: 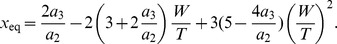 Thus the equivalency is valid when 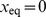, i.e. when
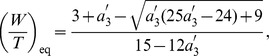
where 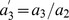. This expression is valid for 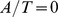, and similar expressions could be obtained for non-zero 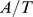 but they become cumbersome. For 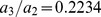 employed in Fig. 5, 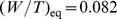. A similar analysis carried out for the antisynchronous state would yield the same value. The equivalency is less accurate at large value of 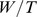 as is also evident from Fig. 5(b, panel for 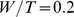). We conclude that negative skewness is in general favorable for antisynchrony and positive skewness is not. Synchrony is stable predominantly when the left PRC jump is negative, or when the right PRC jump is positive. But for sufficiently positive skewness synchrony can be achieved for any 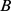 and 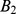, and for sufficiently positive skewness antisynchrony can be made unstable.

### 4. Network Simulations

**Figure 6:**
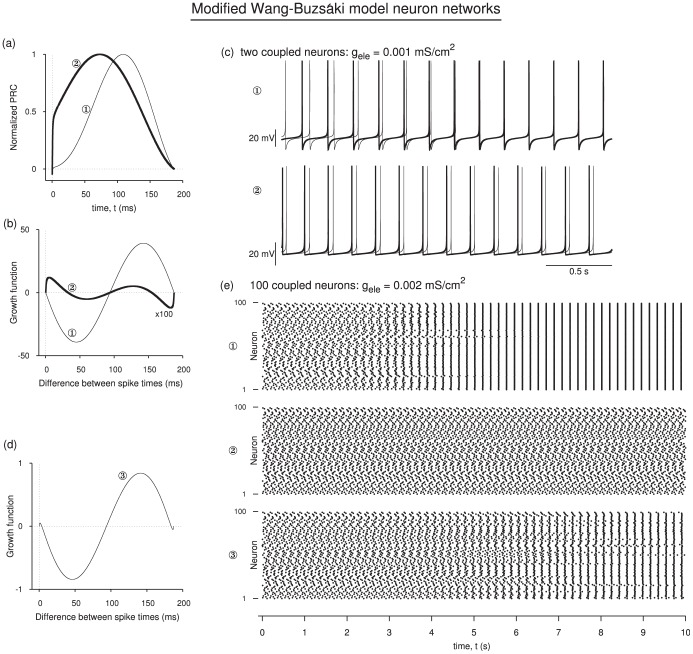
Effect on network behavior emerging from different PRC shapes. Modified Wang and Buzsái model neurons are used to compute PRCs at two parameter sets (a) resulting in a PRC that is nearly symmetric, and another that has very steep rise near small phases. (b) Growth functions corresponding to the PRCs in (a). (c) Voltage time courses of two coupled model neurons corresponding to the parameter sets of the two PRCs in (a). (d) Growth function for a third parameter set resulting in a non-zero phase-locked state near synchrony. (e) Spike times (plotted as dots) of a network of 100 all-to-all coupled model neurons for the three parameter sets corresponding to the curves in (b,d). The non-zero phase-locked state near synchrony leads to prolonged transients, and a jitter in the spike times in the steady state. 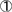: 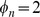, 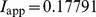 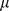
A/cm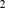. 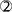: 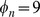, 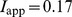 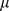
A/cm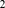. 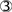: 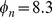, 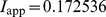 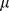
A/cm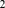. 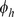 is fixed at 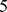 for all the three sets.

We demonstrate here numerically that a PRC with left jump causes synchrony lose its stability using the network of identical modified Wang-Buzsáki model neurons coupled electrically with all-to-all connectivity such that the current 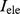 received by 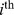 neuron is given by
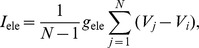
where 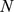 is the number of neurons in the network, and 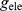 is the strength of electrical coupling. The coupling can be termed weak if in the synchronized state the frequency of the neurons is not significantly altered. Each neuron in the network is made to oscillate by employing an appropriate steady external current such that it’s spontaneous firing rate is about 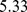 Hz. We test here two parameter sets: 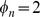, 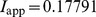 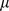
A/cm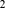, and 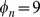, 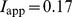 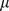
A/cm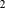. By making the dynamics of the potassium activation faster, we made the nearly symmetric PRC [curve marked 1 in Fig. 6(a)] acquire a sharp rise at zero phase [curve marked 2 in Fig. 6(a)]. The PRC does acquire negative values at early phases below 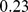 ms, and then rises with a finite slope. But even if this regime was reset such that the PRC gradually approached a positive value at zero phase (corresponding to a left side jump), the results would not differ much. In addition to the sharp rise near zero phase, making the potassium dynamics faster also shifted the peak position of the PRC toward left.

From the theory presented thus far, we expect that both these attributes help destabilize synchrony. The corresponding growth functions are shown in Fig. 6(b) that clearly reveal that left side jump together with the leftward tilt of the PRC caused a destabilization of the synchrony. The antisynchrony remains unstable. A non-zero phase-locked state has acquired stability. Actual solving for the time course using two coupled neurons (N = 2) [Fig. 6(c)] for these two parameter sets verifies this observation.

A network of 100 neurons are coupled electrically starting from initial conditions distributed uniformly on the periodic orbit of the uncoupled neuron, and numerically integrated. Their spike times are shown in Fig. 6(e, top two panels) corresponding to the two parameter sets discussed above. The initial conditions constitute the most inhomogeneous set possible in the network, and the transients decay slowly but gradually and yield either synchronous state for the first parameter set, or an asynchronous state for the second parameter set.

The non-zero phase-locked state is a more sensitive function of the voltage time course and the PRC shape than the synchronous and antisynchronous states. This is because the exact level of the steady state separation between two coupled neurons corresponding to a non-zero phase-locked state is not identical for all parameter changes, unlike synchrony and antisynchrony. Thus their stability is also difficult to compute. But we noted earlier that the case of zero spike width leads to a stable phase-locked state merging with an unstable synchronous state. It is indeed difficult to carry out simulations in such a regime, but a stable phase-locked state can be created near (but not merging with) an unstable synchronous state. In Fig. 6(d) we have used a third parameter set to find that both synchrony and antisynchrony are unstable, but a non-zero phase-locked state very near synchronous state is stable. Carrying out a similar simulation as before using a 100 neuron network [Fig. 6(e, bottom)] reveals that an almost-synchrony may be achieved where the spike times continue to show a small amount of jitter (proportional to the level of phase-locked state), but the network exhibits long transients. The dynamics of such a state depends on a number of factors including how fast a two neuron subnetwork repels a synchronous state (slope of the growth function at zero phase), and how fast such a network approaches the non-zero phase-locked state, and whether there are any other locked states in the system and their stability.

### 5. Special PRCs

#### Flat PRCs

A special case of 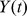 is when the PRC is a flat horizontal line: 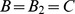. All the eigenvalues corresponding to synchronous and antisynchronous states become zero under this assumption. We can also see the emergence of zero eigenvalues directly from Eqs. 6 and 7. After effecting a transformation of variables in the definition of 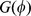, the integral equations expressing 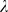 and 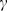 can be written with derivatives of 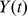 which would become zero under the above assumption. Thus a flat PRC results in neutrally stable synchronous as well as antisynchronous states.

#### Linearly increasing or decreasing PRCs

A special PRC type that is linearly increasing or decreasing from left to right is obtained by setting the PRC maximum to the average of the two jumps: 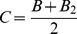
(17)in Eq. 1. First consider stability of synchrony. The two regimes that were treated earlier (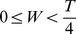 and 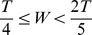) merge into one because the earlier two PRC segments will now become just one due to the special value 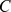. Some of the components 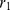, 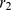, 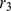, and 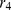 (see Methods) will have different values than 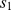, 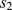, 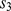, and 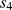 because of altered limits of integration, but their sum will become identical to one another. Substituting for 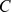 from Eq. 17 in the eigenvalue components 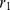, 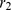, 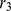, and 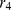, and computing the eigenvalue, we get 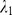 as 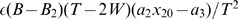 where 
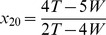
. Solving 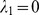 clearly yields the critical curve as: 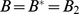. The coefficient of 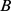 is positive if 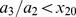 which clearly is the case because it is sufficient for 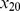 to be bigger than 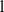 for this relation to hold, but in fact 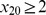 in the range 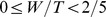 (because 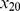 acquires a value of 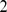 at 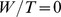 and 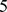 at 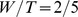, and has a positive slope of 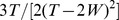 in between). Thus large negative 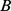 results in 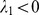 and thus stable synchrony, and large positive 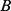 results in unstable synchrony, and the curve 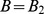 separates the two parameter regions. In summary, the stable region for synchrony is given by



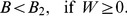
(18)The region of stability did not change with finite spike width although the eigenvalue expression does acquire a dependence on 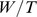 because eigenvalue in either case depends on the difference of the PRC jumps, and the spike width modulates that difference.

Next consider stability of antisynchrony. Consider the case 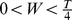. Substituting for 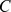 from Eq. 17 in the expression for 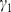 and simplifying, we get 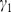 as 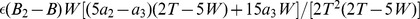 which has clearly a negative coefficient for 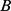. Thus at large negative 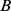, the eigenvalue becomes positive leading to unstable antisynchrony, and at large positive 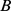 the eigenvalue becomes negative leading to stable antisynchrony. The critical curve that separates these two regimes is obtained by solving 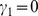 which yields 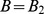. Next in the regime 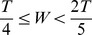, substituting for 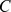 again from Eq. 17 in the expression for 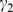, we get the eigenvalue as 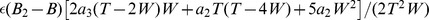 which again has a negative coefficient for 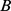 because within the bracketed expression each term is positive due to the current range of 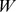. Thus the stability region in the same as above. In summary the region of stability of antisynchrony is given by
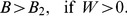
(19)


We can see that at 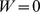, 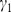 becomes zero independent of 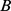 or 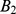 leading to neutrally stable antisynchrony at 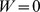. Note that the eigenvalue 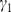 when expanded in Taylor series has a linear dependence on 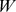 at small 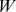: 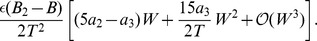



Hence as 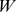 approaches zero, there is no sudden transition from stability to neutral stability. Thus for numerical purposes its stability at 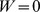 may be considered to follow the condition in Eq. 19. In summary, stable synchrony (Eq. 18) and stable antisynchrony (Eq. 19) exist in complementary parameter regions in 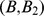 space, and the line 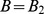 represents critical state where both the locked states merge. It is also clear that the stability regions are independent of spike width and frequency.

## Discussion and Conclusions

The PRC and the voltage are not completely independent. The PRCs are often computed numerically since analytical forms of the PRCs can only be obtained for very few simple models [3]. The standard approach is computing the so called adjoint using averaging technique. In the popular XPPAUT software program [32] this is computed numerically by using a method that uses backward integration technique. If the dynamical system under study exhibits sharp discontinuities such as in adaptive exponential integrate-and-fire model [25], a similar method was recently introduced by Ladenbauer et al. [21]. However, for these two techniques to be employed we must have full knowledge of the differential equations of all the variables in the system. In other words the PRC cannot be completely derived from the voltage time course alone, but is actually derived from the inverse of the derivative of the voltage, and is thus dependent on not only the voltage but all other variables and their derivatives computed on the trajectory.

To compute synchronization properties from experimentally measured PRCs, the above methods are not applicable because differential equation models are not known in general. But the theory of weakly coupled oscillators becomes advantageous if we note that the only two components that are responsible for determining the stability of synchrony and antisynchrony are the shape of the voltage and the shape of the PRC irrespective of all other variables. This gives us an opportunity to comprehensively study the role of each of the shape parameters of the voltage and the PRC on the network behavior without invoking any specific model system. We parameterized the shapes of both the voltage and the PRC. The PRC shape is parameterized by discontinuities and the skewness. And the voltage shape is parametrized by the spike peak, its minimum, threshold level, spike width and period. The Hodgkin-Huxley model voltage time course is only used for the limited purpose of extracting the relationship between the parameters. The information that is extracted is that the voltage may be divided into three segments, and a single spike width parameter 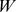 could be used in quantifying both the spike rise and fall profiles. Obviously, not all the PRC and voltage shapes spanned by our study are relevant for a particular given model. But we have presented analytical boundary expressions that can be used in applying the theory to many experimentally determined PRCs and voltages (within the purview of our model), and thus our theory has predictive power. Our study is not the first to incorporate variable parameters in theoretical models. Chow and Kopell [26] and Lewis and Rinzel [12] introduced free parameters of voltage spike width and shape to study the role of them within the context of mostly leaky integrate-and-fire (LIF) like models. But not many experimental results resemble PRCs of the LIF models. Thus we need to parametrize the PRC shapes as well to extend such theories to wider applicability. Our work is an attempt in this direction.

We have investigated the role of sharp jumps in the PRC at its edges on synchronization of electrically coupled neuronal oscillators. The PRC is modeled using a two-piecewise linear function with discontinuous jumps at the edges, i.e. phases corresponding to time 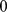 and the period 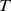. The temporal relationships between the voltage segments are empirically derived from the Hodgkin-Huxley model. But the spike frequency, the spike period, the spike height parameters, and the level of PRC jumps are all freely altered. Analytical boundaries for stability of synchrony and antisynchrony are determined. The main results for small spike widths and frequencies are given by 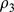 for synchrony and 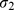 for antisynchrony (relations in Eq. 11 and 13). At large levels of 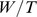, the boundaries are given by 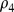 for synchrony, and 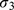 and 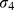 for antisynchrony. We have also studied some special cases of the PRC where its level is either constant, linearly increasing or decreasing.

A positive jump at the left edge contributes to destabilizing the synchronous state, whereas a positive jump at the right edge contributes to its stability. When the depolarizing phase of 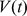 has zero slope (i.e. when 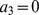), then only the spike upstroke and downstroke play role in deciding the stability, and the eigenvalue for stability is proportional to the difference of the jumps, and more specifically 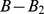; Thus if both edges have equal jumps, then the synchrony is neutrally stable (but the adjacent unstable region has stable non-zero phase-locked states near synchrony), and stability is achieved when the right jump is bigger than the left jump. This relationship holds even for negative jumps. If the depolarizing phase of the voltage has finite slope (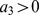), which usually is the case, then it contributes more favorably to synchrony. This leads to the curves 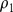 that have decreasing slopes with increasing 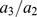 as shown in Fig. 3(a). Large spike width/frequency (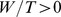) makes it harder to synchronize, and the stable region is moved to lower right as in Fig. 4(a,b).

The jumps at the edges do not directly contribute to altering the stability of antisynchronous state, but they affect the slope of the PRC segments that are connected to them, and those segments alter the stability. But the parameter that affects the stability of antisynchronous state in a sensitive manner is 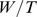. In the absence of spike width antisynchrony must be very rare because it occurs only when the sum of the left and right jumps exceeds twice the PRC level at 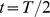 (Eq. 10). It is unstable in the parameter region shown in Fig. 3(a) because the discontinuous jump associated with the voltage at 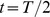 imparts a positive eigenvalue component to the stability and it dominates the magnitude of the negative components from the depolarizing segment of the voltage. When the spike width is finite, the positive eigenvalue contribution from the downstroke (due to its less sharp slope) becomes smaller than the negative eigenvalue contribution from the upstroke (due to its sharper slope) leading to lessening of the destabilizing effect. At appropriate level of frequency (or 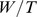), the antisynchrony becomes stable. The overlap between stable synchrony and stable antisynchrony can become more at higher frequencies leading to bistability.

Coupled quadratic integrate-and-fire neuron models at different oscillation frequencies and different spike threshold levels were studied earlier by Pfeuty et al. [23], [24]. Altering the ratio of the spike reset level and the threshold level, (for example from 0.1 to 10 in their Fig. 8 of [23]), transformed the PRC from a state of large jump on the left to a large jump on the right resulting in a transition from stable antisynchrony to unstable antisynchrony. Since we have special parameters to quantify the PRC jumps, in our notation, this means that increasing 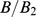 from above 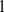 toward zero should make the antisynchrony unstable. This is exactly the case in Fig. 4(a) (refer to the first quadrant). Our boundaries 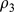 and 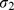 (Eqs. 11 and 13) also quantify the effect of spike width and frequency more explicitly.

The classic leaky integrate-and-fire (LIF) neurons display a PRC (which is 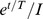 where 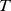 is the period, and 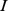 is the applied current) that is indeed discontinuous at either end, but also exponential [12], [26]. Using the PWL methodology we see that 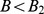 for such a model which ensures stable synchrony (relation in Eq. 18), but relation in Eq. 19 predicts instability for antisynchrony. Our results on the role of skewness (Fig. 5) also indicated that for PRCs that have large positive skewness, antisynchrony is unstable. If the PRC had a diminished, or no response, at early phases, then large PRC skewness could reduce the effectiveness of the spike downstroke (when the spike times are shifted by half period) and cause the antisynchrony become stable.

The piecewise linear approach has been very successful in predicting the stability of synchronous and antisynchronous states. We also note that similar piecewise linear approach when synaptic excitation or inhibition is employed yields quite successful predictions of the role of PRC and voltage shapes on synchrony and antisynchrony [33]. But the non-zero phase-locked states and their stability may not be accurately predicted by a simple two-piecewise linear PRC model because of the fact that the electrical coupling invokes the entire voltage time course. A more elaborate PRC model is needed to address those states. Also, only the first order PRCs are included in the analysis. When higher order PRCs play a significant role in determining the discontinuous jumps, then the approach to and the stability of the phase-locked states may also be affected by their shapes. Finally, strong synaptic input or strong coupling can cause other dynamical solutions that are beyond the scope of the weakly coupled oscillators, such as 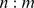 phase-locking that is different from 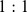, and dynamic spike order switches [34]. Near-synchronous or other non-zero phase-locked solutions could be affected by including the effects of higher-order PRCs [34]. However, weak coupling may still be accurate in predicting the stability of synchronous and antisynchronous states.

## Methods

### 1. Eigenvalue for Synchronous State

#### Case (a) 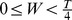.

Consider the case of the spike downstroke occurring before the PRC peak, i.e. 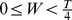. The integral in Eq. 6 can be split into four regimes each of which contains linear segments of 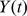 and 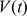. These four regimes contribute to the following four eigenvalue components: 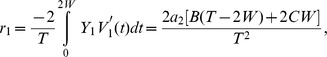


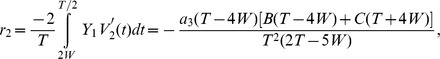


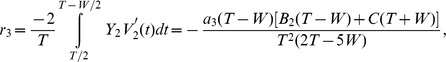


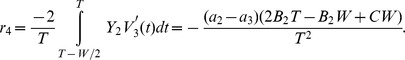



The combined eigenvalue is the sum of the eigenvalue components, and is obtained as.
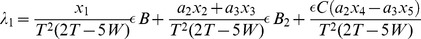
where



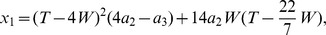


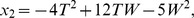


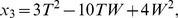


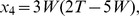


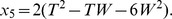



Since 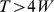 in the present case, we see that 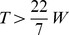, and hence 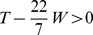 leading to 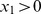. And utilizing the condition in Eq. 3, we conclude that the coefficient of 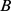 in the eigenvalue expression is positive, and hence, at any fixed value of 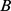 and other parameters, at large positive 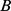 the eigenvalue becomes positive, and at large negative 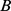, the eigenvalue becomes negative. Hence the synchronous state is unstable at large positive 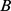, and stable at large negative 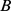. Also since the dependence on 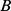 is linear, the derivative of 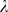 with respect to 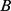 is simply the coefficient of 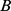 which is positive. Thus the eigenvalue transitions from a negative to a positive value as 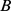 is increased across any critical curve 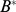. Such critical curve is obtained by solving the equation 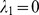 for 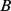, and the stable synchronous region falls below this critical curve. That is the region of stable synchrony is given by 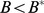 and is expressed in Eq.11.

#### Case (b). 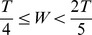


Now consider the case of 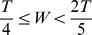. The limits of the four components computed in the previous case are altered because now the spike minimum occurs after the PRC peak position. The four eigenvalue components that contribute to the combined eigenvalue are given below.
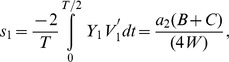


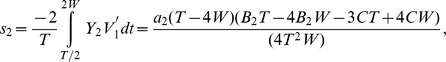


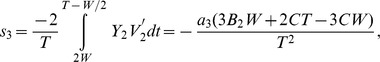


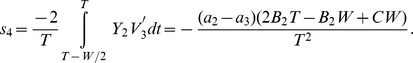



Using the formula in Eq. 6 for these four components, we get the total eigenvalue (we term it 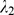) as.
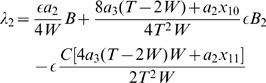
where 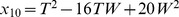, and 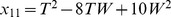. Since the coefficient of 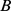 is positive, we directly see that at large negative 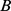, the synchronous state becomes stable because the eigenvalue would become negative, and at large positive 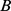 it comes unstable. And the transition across any critical curve 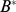 in between is governed by the coefficient of 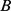 which is positive. And hence the stable synchronous region lies below the critical curve that is obtained by solving the equation 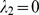: 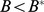 and is as written in Eq. 12.

### 2. Eigenvalue for Antisynchronous State

#### Case (a) 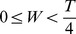.

Finally we find conditions for stability of antisynchronous state. As before, first consider the case 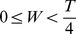. The eigenvalue integral in Eq. 7 can be split into four regimes that have PWL segments, and the four integrals give rise to the following four components: 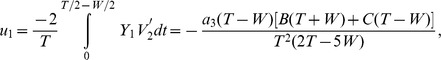


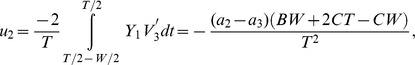


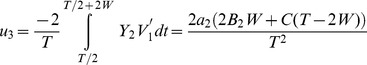


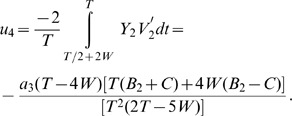



Using the formula in Eq. 7 for these four components, we obtain the total eigenvalue as.
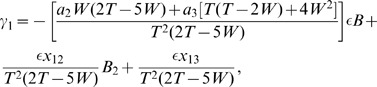
where



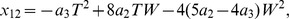


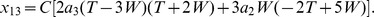



We clearly see (utilizing the condition in Eq. 4) that the coefficient of 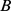 in the eigenvalue expression is negative, and thus for large negative 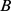, the eigenvalue becomes positive resulting in an unstable antisynchronous state, and for large positive 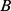 it becomes negative resulting in a stable antisynchronous state. This is exactly opposite to what we obtained above for the synchronous state. And the transition of the eigenvalue as 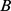 is increased is such that it goes from positive to negative (the derivative of 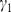 with respect to 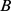 is negative). Thus stable antisynchronous state lies above a critical curve 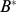 which is obtained by solving the equation 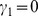 for 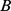: 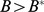 which is expressed in Eq. 13.

#### Case (b) 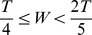.

Next consider the case of 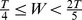. The four eigenvalue components are given by
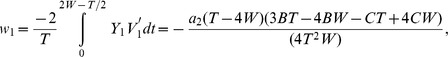


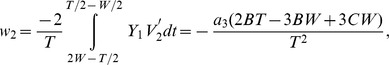


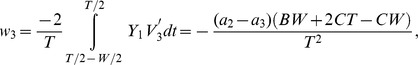


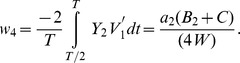



Using the formula in Eq. 7 for these four components, we obtain the total eigenvalue as.
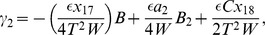
where 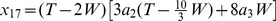, and 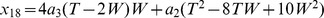. Unlike in the previous case, the coefficient of 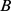 can change sign depending on the value of 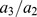. We can directly check that within the present range of 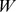, 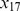 and consequently, the coefficient of 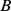 changes sign. The coefficient of 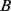 is negative if 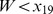 and positive if 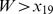, where 
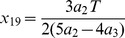
. When 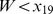, utilizing arguments similar to those used in deriving stability conditions when the coefficient of 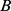 is negative (Eq. 13), we get the region of stable antisynchrony as: 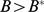 which is given in Eq. 14. When 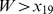, using arguments similar to those used in deriving stability conditions when the coefficient of 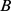 is positive (Eq. 11), we arrive at the region of stable antisynchrony as 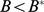 which is given in Eq. 15.
